# Effect of oral antiseptics in reducing SARS-CoV-2 infectivity: evidence from a randomized double-blind clinical trial

**DOI:** 10.1080/22221751.2022.2098059

**Published:** 2022-07-27

**Authors:** Álvaro Sánchez Barrueco, María Victoria Mateos-Moreno, Yolanda Martínez-Beneyto, Elisa García-Vázquez, Alfonso Campos González, Javier Zapardiel Ferrero, Abel Bogoya Castaño, Ignacio Alcalá Rueda, José Miguel Villacampa Aubá, Carlos Cenjor Español, Laura Moreno-Parrado, Verónica Ausina-Márquez, Sandra García-Esteban, Alejandro Artacho, F. Xavier López-Labrador, Alex Mira, María D. Ferrer

**Affiliations:** a ENT and Cervicofacial Surgery Department, Fundación Jiménez Díaz University Hospital, Madrid, Spain; b ENT and Cervicofacial Surgery Department, Villalba General University Hospital, Collado Villalba, Spain; c Department of Dental Clinical Specialties, School of Dentistry, Madrid Complutense University, Madrid, Spain; d Department of Dermatology, Stomatology and Radiology, University of Murcia, Murcia, Spain; e Murcian Institute of Biosanitary Research (IMIB), Murcia, Spain; f Infectious Diseases Unit, Virgen de la Arrixaca University Clinical Hospital, IMIB, Murcia, Spain; g Microbiology Department, Fundación Jiménez Díaz University Hospital, Madrid, Spain; h Villalba General University Hospital, Collado Villalba, Spain; i Microbiology Service, Murcian Institute of Biosanitary Research, Virgen de la Arrixaca University Clinical Hospital, Murcia, Spain; j Department of Dentistry, European University of Valencia, Valencia, Spain; k Genomics & Health Department, FISABIO-Public Health Foundation, Valencia, Spain; l Department of Microbiology and Ecology, Medical School, University of Valencia, Valencia, Spain; m CIBER in Epidemiology and Public Health (CIBERESP), Instituto de Salud Carlos III, Madrid, Spain

**Keywords:** SARS-CoV-2, saliva, COVID-19, infectivity, mouthwash

## Abstract

Background: *In vitro* studies have shown that several oral antiseptics have virucidal activity against SARS-CoV-2. Thus, mouthwashes have been proposed as an easy to implement strategy to reduce viral transmission. However, there are no data measuring SARS-CoV-2 viability after mouthwashes *in vivo*. Methods: In this randomized double-blind, five-parallel-group, placebo-controlled clinical trial, SARS-CoV-2 salivary viral load (by quantitative PCR) and its infectious capacity (incubating saliva in cell cultures) have been evaluated before and after four different antiseptic mouthwashes and placebo in 54 COVID-19 patients. Results: Contrary to *in vitro* evidence, salivary viral load was not affected by any of the four tested mouthwashes. Viral culture indicated that cetylpyridinium chloride (CPC) significantly reduced viral infectivity, but only at 1-hour post-mouthwash. Conclusion: These results indicate that some of the mouthwashes currently used to reduce viral infectivity are not efficient *in vivo* and, furthermore, that this effect is not immediate, generating a false sense of security.

**Trial registration:** ClinicalTrials.gov identifier: NCT04707742..

## Introduction

The Coronavirus Disease 2019 (COVID-19) pandemic caused by SARS-CoV-2 has already infected more than 530 million people and has caused almost 6,3 million deaths globally [1] in 2 years. In addition to vaccination, therapeutic strategies must also be pursued, as they are essential to stop the severity of the disease and the spread of the virus.

Transmission of the virus occurs by respiratory route (aerosols and respiratory droplets) and by contact with contaminated surfaces followed by contact with nasal, oral or ocular mucosa [2]. Transmission can happen in pre-symptomatic, symptomatic or asymptomatic patients [3]. Therefore, prevention practices such as the use of masks, hands’ hygiene and social distancing remain the key pillars of public infection control to avoid contagion.

The detection rate of virus in the saliva of COVID-19 patients reaches 91.7% [4] thus recognizing saliva as a valid substrate for the detection of SARS-CoV-2 [5]. This also implies that direct or indirect contact with saliva represents a risk of COVID-19 transmission. This is very relevant for the general population but especially risky for professionals with direct access to the oral cavity such as dentists, ENT (Ear Nose and Throat) and cervicofacial surgery specialists, or maxillofacial surgeons. For these reasons, various health authorities have suggested the use of antiseptic mouthwashes by patients as an infection control measure prior to the procedure [6]. However, the above recommendations are not supported on evidence-based clinical data.

There is a considerable number of publications in the literature studying the *in vitro* effect on viral load of different oral antiseptics which are part of mouth rinses [7–14]. However, there is still a disparity of results between studies, even contradicting each other's postulates. In addition, there are few clinical studies available, with strong design flaws such as lack of a control group or pilot studies with extremely low samples sizes [15–18]. In addition, all these clinical studies assess viral load reduction based on RT-qPCR results, without taking into account the effect of the mouthwash on viral infectivity. For example, in a recent clinical study, none of the four mouthwashes tested reduced total viral load as measured by RT-qPCR in saliva, but it is unknown if any of the residual viral genome equivalents detected are infectious [19]. Culturing SARS-CoV-2 from saliva samples has proven to be technically challenging [17], and therefore it has been difficult to evaluate viral infectivity in vivo after the use of oral antiseptics.

It is therefore necessary to build on established knowledge to demonstrate the actual effectiveness of different mouthwashes on viral load, including their effect on viral infectivity. In the current manuscript, the results from a randomized, double-blind, five-parallel-group, placebo-controlled clinical trial where the effect of four different oral antiseptics is evaluated both by RT-qPCR (total viral load) and for the first time by infection of the saliva samples in cell cultures (infectious viral load). Mouthwash products and concentrations were selected based on previous efficacy studies in *in vitro* studies [7–14] and products’ commercial availability, in order to facilitate direct use during the pandemic. Thus, the purpose of our study was not to compare equivalent concentrations of antiseptics, but to test efficacy for already-developed formulations. The results were compared to a placebo group where participants performed the same mouthwash with water, establishing a comprehensive evaluation of the efficacy of the different compounds tested, and therefore their potential to reduce SARS-CoV-2 transmission.

## Results

**Figure 1.**
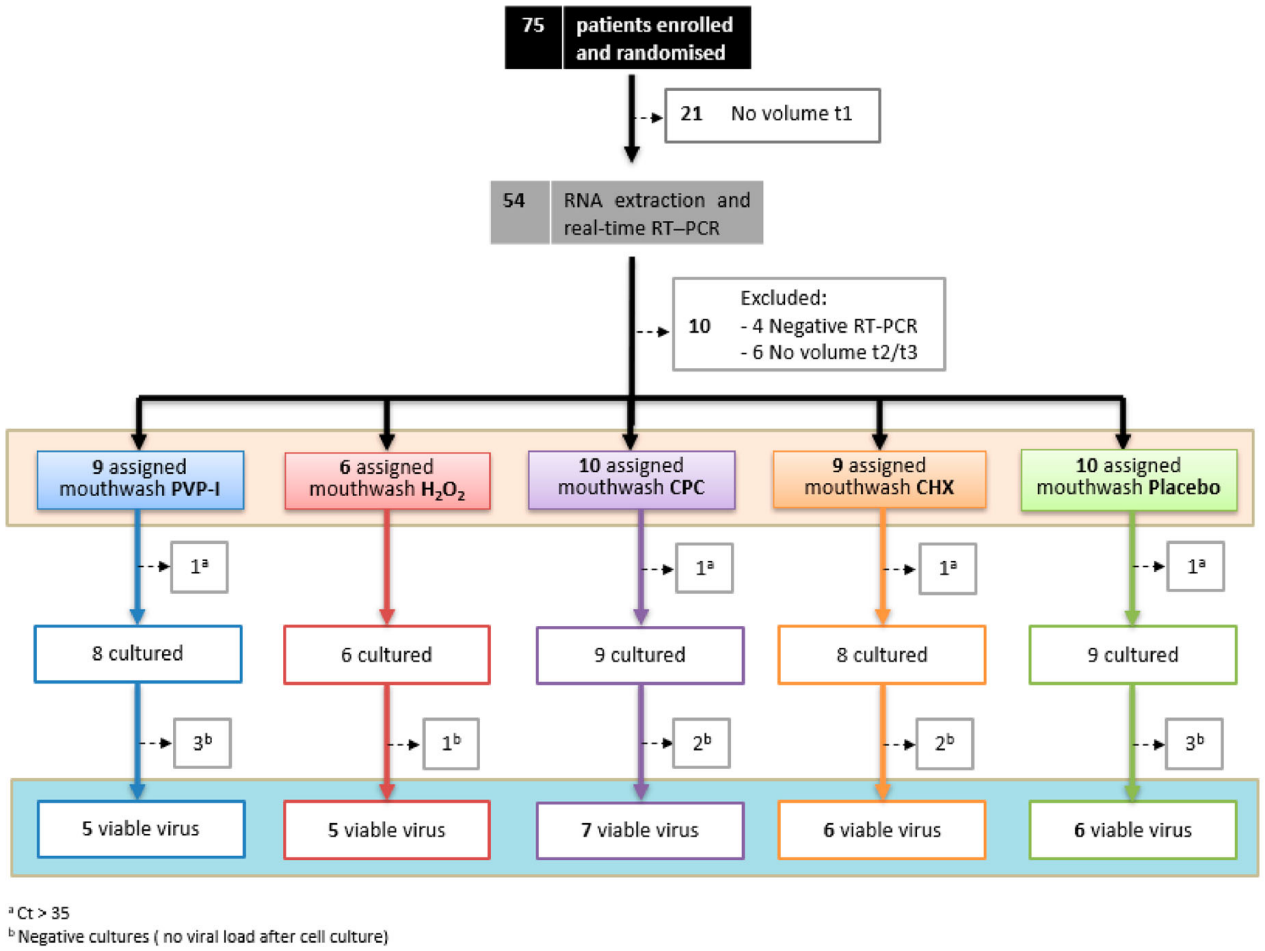
Trial profile. Diagram showing the number of patients enrolled and randomized, excluded for not having sufficient saliva volume or negative to RT-qPCR in the baseline samples, and the total evaluated patients in which total viral load (orange square) and viable (infective) viral load (blue square) were determined. The numbers of inpatients assigned to each of the different treatment groups are represented inside boxes of different colours. PVP-I (povidone-iodine) in blue, Hydrogen peroxide (H_2_O_2_) in red, CPC (cetylpyridinium chloride) in purple, CHX (chlorhexidine) in orange, and Placebo (distilled water) in green. t1: basal time point. ^a^ Number of patients excluded from the cell culture assays for low salivary viral load (Ct value > 35 in the RT-qPCRs in the baseline saliva sample). ^b^ Number of patients without detectable viral load after cell culture in the baseline saliva sample.

From the 75 enrolled and randomized patients, 21 had to be excluded from the analysis because of insufficient baseline saliva volume to perform quantifications (Figure 1).

**Figure 2.**
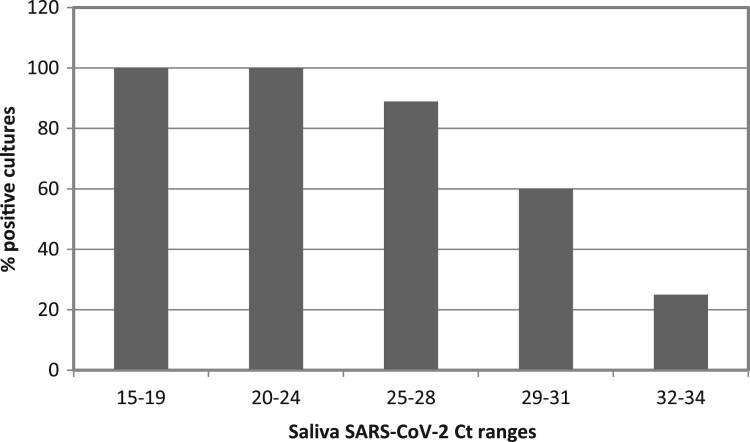
Positive viral culture as a function of salivary viral load. Bars represent the percentage of SARS-CoV-2 positive viral culture of in saliva samples from COVID-19 inpatients. Saliva samples were incubated in Vero-E6 cells for 1 h to allow viral adsorption and then replaced by fresh culture medium. At day 5 post-infection, viral replication was measured by CPE reading and SARS-CoV-2 RNA quantitation in the culture supernatant. A culture was considered positive by RT-qPCR Ct values < 37 in day 5 post-infection supernatant (equivalent to ≥ 2 × 10^3^ SARS-CoV-2 copies per mL). The number of patients in each Ct range was: n = 3 in Ct range 15-19, n = 10 in Ct ranges 20–24 and 29–31, n = 9 in Ct range 25–28 and n = 8 in Ct range 32–34.

After RNA extraction and real-time RT-qPCR quantification was performed for all 162 samples from the remaining subjects, 10 volunteers were discarded due to undetectable viral load or lack of sufficient volume in saliva samples after rinsing to conduct the viability assays in cell lines. Thus, the viral load in saliva was quantified in forty-four individuals assigned to one of the five study groups, with a sample size of 6–10 patients per group. At this point, a maximum threshold value of Ct in the basal saliva samples prior to rinsing was established to select the samples to be tested in the viability assays in cell cultures. Based on the previous results of other groups on the correlation between successful isolation of virus in cell culture and Ct value of quantitative RT-qPCR, such as those of La Scola et al. [20], four individuals were excluded for obtaining a Ct value in basal saliva higher than 35. The results showed that all samples with a basal saliva Ct lower than 25 had successful viability in cultures. In contrast, as the Ct value in the saliva sample increased, the percentage of positive cultures gradually decreased, being less than 30% in those samples with Cts greater than 31 (Figure 2).

Of the 135 saliva samples from 40 patients (between six and nine inpatients per treatment group) tested in the cell culture assays, SARS-CoV-2 could be isolated in cell culture from a sample size of 5–7 individuals per group, which were used for paired statistical comparisons.

**Figure 3.**
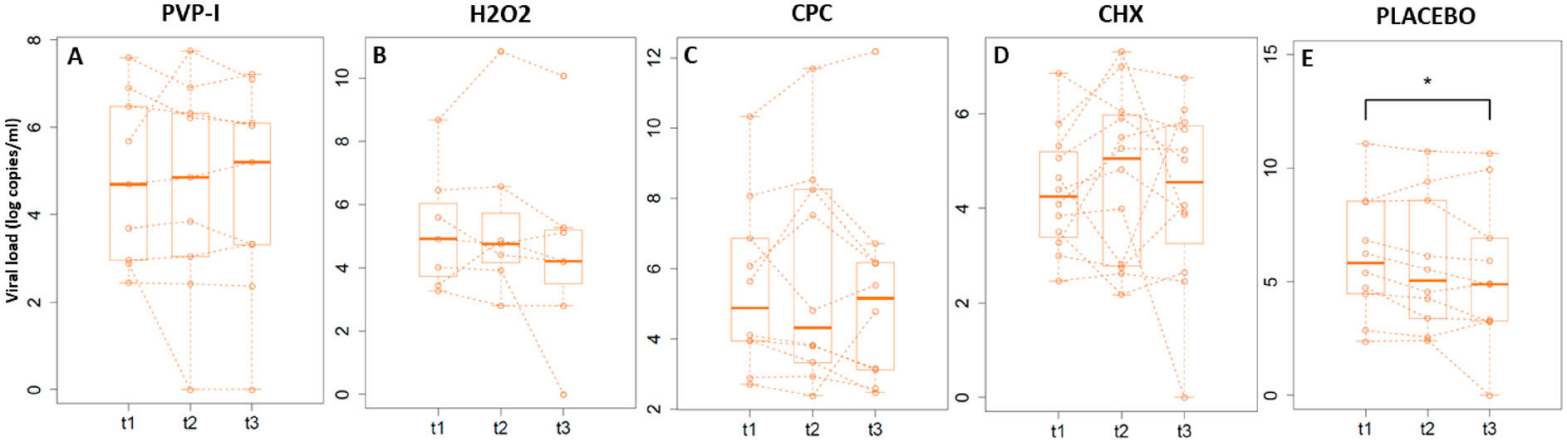
Salivary viral loads. Box plots represent the median values of viral loads in log copies per mL of saliva measured by RT-qPCR for basal saliva (t1), 30 min (t2) and one hour (t3) after the oral rinse for each treatment group: PVP-I (povidone-iodine), Hydrogen peroxide (H_2_O_2_), CPC (cetylpyridinium chloride), CHX (chlorhexidine) and PLACEBO (distilled water). The dotted lines join the values for the same patient through time. Different y-axis scales are used, for clarity. * Wilcoxon paired test (*p* value = .048).

After randomization, the basal salivary viral load did not differ between groups (*p*-value between .12 and .67). None of the four tested mouthwashes reduced the total viral load in saliva either at 30 minutes after rinsing or at 1 hour (Figure 3). Unexpectedly, a significant decrease in the mean values of viral load in saliva was detected 1 hour after rinsing with water (*p*-value: .05).

**Figure 4.**
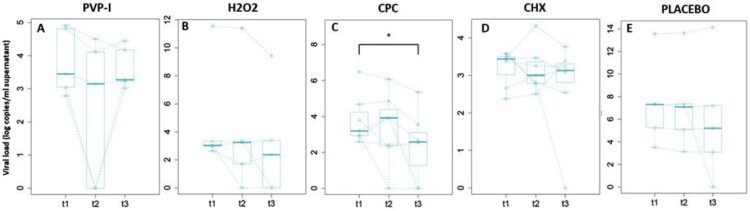
SARS-CoV-2 genome copies/mL in supernatants from day 5 virus culture in Vero-E6 cells (median values in log copies per mL of culture supernatant measured by RT-qPCR). Box plots are represented for each treatment group (PVP-I (povidone-iodine), Hydrogen peroxide (H_2_O_2_), CPC (cetylpyridinium chloride), CHX (chlorhexidine) and PLACEBO (distilled water)) at each time point (t1 for basal and t2 and t3 for 30 and 60 min after the mouthwash, respectively). The values for each patient are linked over time by dotted lines. Different y-axis scales are used, for clarity. * Wilcoxon paired test (*p* value = .015).

Next, we evaluated saliva samples for SARS-CoV-2 infectivity by virus culture in Vero-E6 cells for five days. We used SARS-CoV-2 genome copies/mL in the day 5 culture supernatant as a proxy for the initial amount of infectious virus in the saliva samples. The results for each of the five study groups at each saliva collection time-point are shown in Figure 4.

In this case, a significant decrease of 1.5 log genome copies/mL of culture supernatant was observed in the mean number of infectious viruses in the group treated with CPC (Group C) 1 hour after rinsing with respect to the basal saliva prior to rinsing (*p*-value: .02). Specifically, a mean of 3.8 log genome copies/mL of culture supernatant were obtained in the basal sample, compared to 2.3 log genome copies/mL of culture supernatant at 1 hour after rinsing with CPC. The decrease in the mean amount of infectious viruses observed 1 hour after rinsing with CPC corresponds to a reduction in viral infectivity of 97.16%. No significant differences in the amount of cell-culture recovered virus were found in saliva taken at 1 hour after rinsing for the other treatments. In samples taken at 30 minutes after rinsing, no significant differences were observed for any of the mouthwashes, including CPC, although a trend towards lower values in the amount of cell-culture recovered virus was detected in PVP-I patients (Group A) at 30 min after rinsing as compared to cell-culture recovered virus from baseline samples prior to rinsing (*p*-value: .06). Thus, although the different mouthwashes did not affect the SARS-CoV-2 viral loads in saliva, samples taken 1 hour after rinsing with CPC showed a significantly reduced levels of cell-culture recovered virus in our in vitro infection system.

The proportion of men in each group was higher than women, the latter ranging between 40 and 45%, except in group A where 54% were women (Table 1). The mean age of the patients enrolled for the five groups was between 55 and 69 years. The mean number of days since the last nasopharyngeal PCR performed in the hospital and the number of days of symptoms for each group was between 3–4 days and 5–7 days, respectively.

**Table 1.** Clinical variables.

		A Povidone-iodine (PVP-I) n = 9	B Hydrogen peroxide (H_2_O_2_) n = 6	C Cetylpyridinium Chloride (CPC) n = 10	D Chlorhexidine (CHX) n = 9	E Placebo (H_2_O) n = 10
Sex	Female	54%	45%	40%	43%	43%
	Male	46%	55%	60%	57%	57%
Age (years)		65 (42-82)	62 (41-85)	69 (48-90)	55 (25-90)	60 (35-88)
Days from last PCR		3 (0-14)	3 (1-11)	3 (1-10)	4 (0-14)	3 (0-12)
Symptom days		5 (2-9)	6 (1-11)	6 (2-11)	7 (0-16)	6 (0-11)

Data show the percentage of males and females in the participants, their mean age, the days from the positive SARS-CoV-2 PCR nasopharyngeal test performed at the hospital and the days since the appearance of symptoms related with COVID disease, for each treatment group.

## Clinical variables

There was no statistically significant correlation between baseline salivary viral load or nasopharyngeal viral load with age, sex, or days since the last SARS-CoV-2 RT–PCR result. Instead, a statistically significant negative correlation was found between days of symptoms and basal saliva viral load (Spearman’s correlation coefficient: −0.344; *p*-value: .03). Statistically significant correlation was also found between basal salivary Ct values and nasopharynx Ct values (Spearman's correlation coefficient: 0.44; *p*-value: .004). Finally, a significant correlation was found between salivary Ct values and Ct values from viral culture supernatants (Spearman's correlation coefficient: 0.651; *p*-value: .0001), and between the nasopharyngeal Ct values and Ct values from viral culture supernatants (Spearman's correlation coefficient: 0.504; *p*-value: .007).

## Discussion

We have analysed the *in vivo* effect of four mouthwashes, versus placebo, on the salivary SARS-CoV-2 viral load (viral particles per mL of saliva) and viral infectivity (SARS-CoV-2 replication in cell culture). This is the first time to our knowledge that SARS-CoV-2 infectivity after antiseptic mouth rinse has been studied on a cell culture system. Specifically, the effect of four individual mouthwashes, including PVP-I 2%, H_2_O_2_ 1%, CPC 0.07%, CHX 0.12% and distilled water as a placebo group, was analysed. Saliva was reaffirmed as a valid substrate for the study of SARS-CoV-2 viral load [5], especially when few days have passed from the appearance of the symptoms. When viral load was high (salivary RT-qPCR Ct values < 25) infection in a Vero-E6 cell line system was successful in all cases, indicating that this is the limiting factor to efficiently culture SARS-CoV-2 from saliva specimens. From a public health perspective, it is important to keep in mind that, at least in our in vitro system with Vero-E6 cells, modest salivary Ct values of 30 still corresponded to over 50% of the viral particles being able to infect. This implies that low-sensitivity SARS-CoV-2 tests could fail to detect cases with infective potential. Interestingly, Ct values in the nasopharynx were significantly correlated with those obtained in cell culture supernatants from basal saliva samples, confirming viral culture from saliva samples as a valid proxy of viral infectivity. There was a significant negative relationship (Pearson’s correlation coefficient 0.344) between days from initiation of symptoms and salivary viral load, indicating that saliva viral load decreased correlating with the number of days from symptom onset, in line with previous reports of negative viral detection in saliva when RT–PCR in nasopharyngeal samples was still low-positive [12].

Contrary to the extraordinary results obtained *in vitro* for different oral antiseptics [8–12,21] our data show that the capacity of the same mouthwashes to reduce viral load or infectivity *in vivo* is modest. A significant reduction in salivary viral copy numbers of 1.5 log and a 97.16% reduction in viral infectivity were obtained only for CPC, and only 1 hour after oral rinsing, suggesting that the effect is not immediate. In addition, there was a trend towards a reduction in infectivity 30 minutes after rinsing with PVP-I, encouraging future testing of formulations including these compounds.

Saliva viral load has been proposed as a predictor of disease severity, given that salivary viral load has been shown to be significantly higher in patients with COVID-19 risk factors. A comparative study of viral load between endotracheal aspirate and saliva samples revealed that patients with COVID-19 had the highest salivary viral load during the first week after symptom onset [22], and then progressively decreased. This could probably explain the rapid progression of the pandemic and justify the promulgation of the use of antiseptics as a preventive health measure [23].

The vast majority of studies testing mouthwash efficacy against coronaviruses published have been conducted *in vitro* [7]. In these, 15–60 s contact with different oral antiseptics resulted in strong virucidal effects ranging from one to four orders of magnitude reduction in viral infectivity [24–26]. In fact, recent studies hypothesize that the organic components of mouthwashes may alter viral membranes or act on viral proteins [8]. However, these protein alterations can lead to cytotoxicity, which affects not only viable viruses but also cultured cells used in the infection studies, limiting the range of concentrations at which the antiseptics can be properly evaluated and constraining the interpretation of results. It is also important to keep in mind that active ingredients in the oral cavity are rinsed and reduced in concentration by saliva clearance [27], and that the exposure time of the virus to a given compound in vivo will vary depending on its substantivity (retention time) and the individual’s salivary flow. Another factor unaccounted for in vitro is the mechanical effect inherent to rinsing. This shearing effect must lead to a rupture of cells and viral-cell junctions, which would ultimately facilitate the virucidal effect. In addition, saliva is a complex fluid with dozens of proteins and glycoproteins where the effect of antiseptics will be modified, and where present bacteria will also bind to the active ingredients, limiting the levels available to interact with the virus. We propose that a combination of these factors contribute to the lack of consistency between in vitro and in vivo results, with mouthwashes being a priori more effective in the former and these results not always reproducible in vivo.

Our results therefore recommend clinical testing of oral antiseptics with promising in vitro results before recommending usage guidelines. Recently, a systematic review of in vitro studies confirmed the high virucidal capacity of PVP-I [12]. In our study, this shows a slight trend, with a decrease in viral load in saliva of less than 0.5 logs at 30 minutes, and therefore future studies should evaluate if this compound provides an immediate virucidal effect that is lost with time, as recently reported for the antiseptic Linola sept, which showed a maximum effect right after the application [28].

Muñoz-Basagoiti et al. specifically studied the effect of CPC in vitro, showing that it reduced SARS-CoV-2 infectivity by altering the integrity of the viral envelope [9]. This activity was effective for different SARS-CoV-2 variants, reducing the viral infectivity (TCID50/mL) by at least three logs, so it was postulated that CPC rinses could represent a cost-effective health measure to reduce SARS-CoV-2 transmission. Thus, rinsing for 1–2 minutes should be sufficient to effectively decrease virus infectivity in saliva, especially during the first 2 weeks after infection, when higher viral titres are detected and individuals are more infectious [9]. In the present study, however, we found the significant effect of reducing viral viability occurring 1 hour after rinsing, in agreement with the long substantivity of this compound in the oral cavity, and suggesting a cumulative effect [19]. Similarly, Koch-Heier et al. [10], studied the in vitro effect of mouthwashes individually (0.05% CPC, 0.1% CHX solution or 1.5% H_2_O_2_), a non-commercial combination (0.05% CPC and 0.1% CHX) or commercial formulas such as ViruProX^®^ (0.05% CPC + 1.5% H_2_O_2_) or BacterX^®^pro (CHX 0.1%, + CPC 0.05% + fluoride 0.005%). H_2_O_2_ and CHX alone had no virucidal effect against SARS-CoV-2, but CPC alone or combined with CHX was associated with a significant reduction of infectious virus [10]. Thus, future work should study the efficacy of different compound combinations that could provide synergistic effects. For example, in the series of Huang et al. [29], CHX 0.12% was studied in hospitalized patients with COVID-19. SARS-CoV-2 RNA (measured by RT-qPCR) was cleared from the oropharynx in 62.1% of patients using CHX 0.12% as a mouthwash. Thus, it was inferred that CHX used as a mouth rinse and subsequent oropharyngeal spray may have significant effects in controlling the spread of disease [29]. Similarly, Costa et al. found that CHX 0.12% decreased viral load at 5 and 60 minutes after rinsing [30]. This could not be corroborated in our study, where CHX had no significant effect on total viral load or viral viability measured in cell cultures. Thus, the combination of CHX with other components in the different commercial mouthwash compositions may provide different final effects, whose action mechanism is not yet understood. Similarly, in the series of Eduardo et al. [31], the effect of CPC 0.075% + Zinc Lactate 0.28%, H_2_O_2_ 1.5%, CHX 0.12%, H_2_O_2_ 1.5% + CHX 0.12%, and placebo (distilled water) was studied in 60 SARS-CoV-2-positive patients. The virucidal effect of the mouth rinse was assessed as a function of the change in salivary viral load by RT-qPCR. CPC + Zinc lactate and CHX mouth rinses resulted in significant changes in Ct values (an increase between 1 and 3 PCR cycles) in saliva up to 60 min after rinsing. The H_2_O_2_ mouth rinse resulted in a significant effect 30 minutes after rinsing. However, none of their results were corroborated in our series, except for CPC. Other studies such as Seneviratne et al. [15], studied the in vivo effect of CHX 0.2%, CPC and PVP-I in 16 patients. Although no significant differences in viral load were found relative to baseline values, the control group (n = 2), using water, did show an increase in viral load with time. The series published by Elzein et al. [32], showed that rinses with CHX 0.2% or PVP-I 1% reduced viral load (again measured by RT-qPCR) within 5 minutes of rinsing, with an increase in Ct values between four and five PCR cycles, which would theoretically correspond to 1.5 log difference in viral load. Thus, available results from clinical studies appear to be inconsistent with a lack of control (placebo) groups in many studies, but in overall suggesting a moderate effect of some mouthwashes, with a considerably lower effect than that observed when tested under in vitro conditions.

Based mainly on the outcome of the available *in vitro* studies at the initial stages of the pandemic, the use of mouth rinses has been empirically recommended as a protocol prior to dental and several medical procedures in order to reduce the viral load and the possibility of viral transmission [33,34]. Although some studies of the virucidal effect of mouth rinses *in vivo* have recently been published [15,29–32], none of them complement the study of RT-qPCR with assessment of SARS-CoV-2 infectivity by viral cell culture from the collected saliva samples. Our data underscores the need for rigorously evaluating viral infectivity in cell culture after mouthwash exposure, and not relaying only in the detected salivary, which is a measure of both infectious and non-infectious virus.

Limitations of our study include the inclusion of only inpatients, which allowed for the collection of saliva samples with immediate freezing, but reduced the range of patients tested. In addition, the limited sample size in our study should be expanded in future work in order to increase statistical power. It was also surprising to find a low but significant decrease in total viral load in the placebo group (Figure 3). A possibility is that the mechanical forces during the rinse release viral particles more effectively with water or that distilled water affects viral viability through osmotic pressures. However, many *in vitro* studies use distilled water as control, and they did not detect a virucidal effect [35]. In addition, we only observe a reduction in the total viral load but not in the infection essay, suggesting that distilled water could affect viral lipids reducing attachment but it is unlikely that it affects viral protein stability and therefore has no effect on viability. Viral culture in monkey Vero-E6 lines was used to asses SARS-CoV-2 infectivity because it is the most widespread and tested *in vitro* system for culturing coronaviruses [8,10,13,14]. Nevertheless, new protocols with human lung cells are being developed and improved (see, for example, Schütz et al. [36]) and future studies should focus on human cell lines as a more realistic infection model.

In summary, our results and, other published available to data indicates that the use of mouthwashes containing CPC or PVP-I to reduce SARS-CoV-2 load in the oral cavity should be further explored. Despite that, the effects detected in reducing viral infectivity in saliva (and therefore decreasing SARS-CoV-2 infectivity) are modest and always below two logs in the best cases, and our study shows that 1 hour after the mouth rinse may be required for a significant antiviral effect. It is therefore a concern that some mouthwashes currently used may confer a false sense of security while providing limited protection from viral transmission. In addition, the lack of an immediate effect should be further evaluated before specific application directions are proposed. Thus, we hope that the current study assessing the effects of mouthwashes in viral infectivity in vitro stimulates further research in the use of oral antiseptics against viral infections, underscoring the need for keeping other safety measures in clinical settings until the clinical efficacy of mouthwashes properly established.

## Methods

### Study design

The in vivo effect of several antiseptics on viability of SARS-CoV-2 in saliva samples of COVID-19 patients was evaluated by a randomized, double-blind, five-parallel-group, placebo-controlled trial (ClinicalTrials.gov Identifier: NCT04707742).

The study was performed in three different hospitals: Fundación Jiménez Díaz University Hospital (Madrid, Spain), Villalba University General Hospital (Collado Villalba, Spain) and Virgen de la Arrixaca University Hospital (Murcia, Spain), after approval by the Ethical Committee of the Fundación Jiménez Díaz University Hospital on 2020/11/24th with code EO095-20_FJD-HGV-HIE.

### Patients

All the participants were adult inpatients (age >18 years) diagnosed and hospitalized with SARS-CoV-2 positivity, developing COVID-19 disease. Every patient provided their voluntary written or oral consent to participation and met inclusion criteria, namely: a positive result for SARS-CoV-2 test of a nasopharyngeal sample in the previous 10 days and the ability to perform a mouthwash and donate saliva samples. Only patients with nasopharyngeal samples showing RT-qPCR SARS-CoV-2 E-gene Ct values lower than 30 were selected, in order to ensure further cell culture. Exclusion criteria were the use of an antiseptic mouthwash for 48 h before the start of the study, patients receiving antiviral drugs or participation in a COVID-19 research study testing experimental drugs and any allergy or hypersensitivity to the mouthwashes’ components.

Epidemiological data was collected including age, gender and time of symptoms’ onset.

### Randomization and masking

Each participant was consecutively assigned a code consisted of a patient number and a letter corresponding to one of the five study treatments groups (A, B, C, D and E) from a previously randomly generated table that was unknown to the research team who performed RNA extraction, RT-qPCR and analysed the data. Identical tubes with the same volume for both mouthwashes and placebo were used to achieve masking.

### Mouthwashes and procedures

The five different study rinses were randomized into five treatment groups as follows: Group A (povidone-iodine, PVP-I, 2%), Group B (hydrogen peroxide, H_2_O_2_, 1%), Group C (cetylpyridinium chloride, CPC, 0.07%), Group D (chlorhexidine, CHX, 0.12%) and the placebo group, Group E (distilled water). The concentrations of mouthrinses A (Betadine^©^ Bucal 100 mg/mL) and B (Oximen^©^), were adjusted to those indicated by the manufacturer minutes before rinsing, diluting commercial formulas with distilled water (in group A, commercial PVP-I 10% was diluted to 2%; and in group B, hydrogen peroxide 3% was diluted to 1%). Group C (Vitis Xtra Forte^©^) and D (Clorhexidina Dental PHB^©^) rinses were ready to use in their commercial formulas. Distilled water was used as placebo because *in vitro* studies with oral antiseptics showed that distilled water had no virucidal effect on SARS-CoV-2 [35], because the base component of all tested mouthwashes was water and because in the cases where the mouthwash had to be diluted prior to use for its final working concentration (povidone iodine and hydrogen peroxide), distilled water was also used.

**Figure 5.**
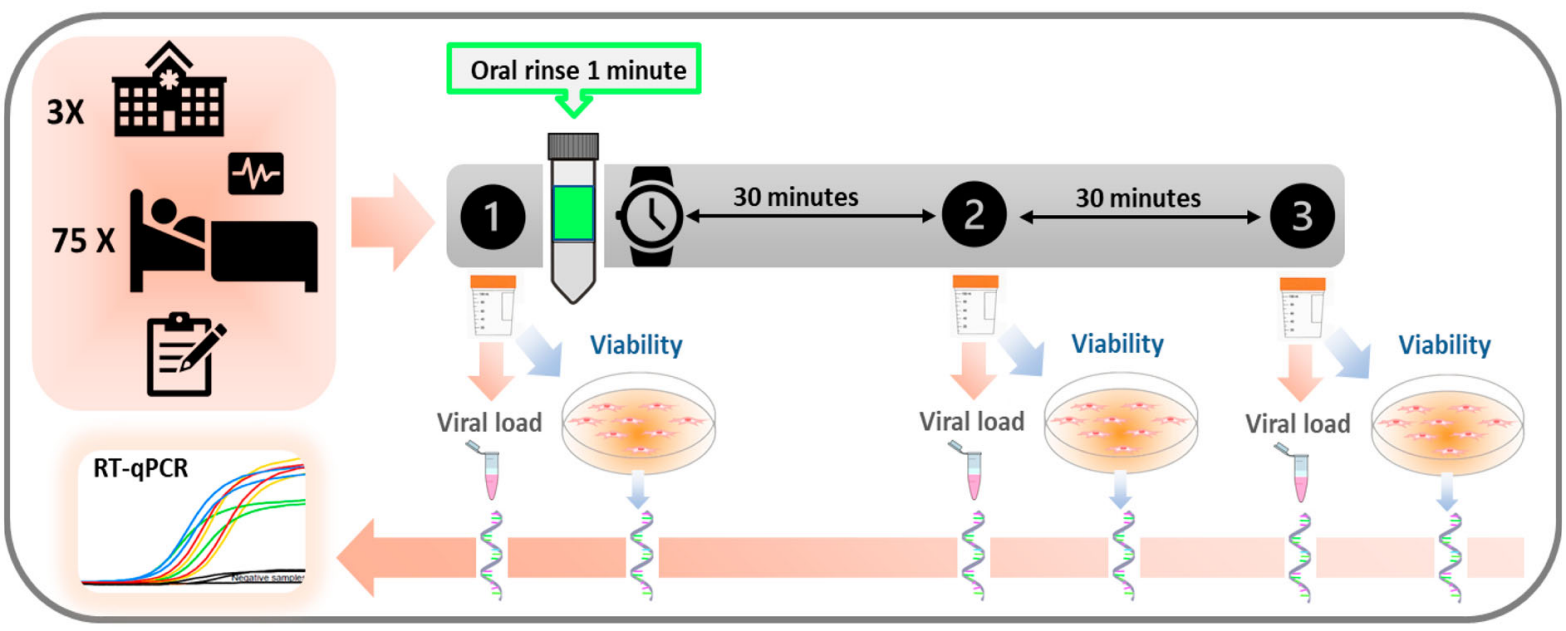
Outline of the clinical trial protocol. From top to bottom, the upper left box represents: the number of hospitals involved in this multicenter study, the number of enrolled and randomized inpatients and the registry of their clinical variables. On the right, the timeline protocol for collecting the three non-stimulated saliva samples is illustrated, before (1) and 30 min and one hour after (2 and 3, respectively) the one-minute oral rinse with the respective mouthwash. All samples were taken to the laboratory where each saliva sample was divided into two aliquots, one to determine the viral load per mL of saliva, by means of its RNA extraction and subsequent RT-qPCR (reverse transcription-quantitative polymerase chain reaction), and the other to assess viral infectivity in Vero-E6 cell culture, by detection of cytopathic effect (CPE) and viral replication (RNA extraction and RT-qPCR of the virus culture supernatant).

Three samples of unstimulated saliva were donated by each participant: one baseline prior to rinsing and the other two at 30 minutes and 1 hour after rinsing, respectively (Figure 5). For each sample collection, the patients were asked to donate at least 2 mL of unstimulated saliva in a sterile plastic tube millimetre using the drooling technique, avoiding spilling secretions of respiratory origin.

Participants were not allowed to chew gum, smoke, brush their teeth or drink anything but water, for 1 hour prior to sample collection and until the end of the intervention.

After the first sample collection (basal saliva, time point t1), the patient was asked to rinse with 15 mL of the mouthwash for 1 minute, trying to get the mouthwash to all teeth, palate, gums and tongue, without swallowing or gargling. Half an hour (time point t2) and 1 hour after (time point t3) rinsing respectively, the other two saliva samples were taken. Immediately after each collection, every sample, previously labelled with the patient code and time point, were introduced in an airtight bag and kept at −80°C until transfer to the laboratory. For sample analysis, all secondary containers of airtight bags with the three saliva samples of each patient were introduced in a rigid box with dry ice according to UN3733 standards and sent to the laboratory (FISABIO-Public Health) by courier service. Once in the laboratory, all samples were stored at −80°C.

### Nucleic acid extraction and real-time RT–qPCR

Saliva samples were processed at the biosafety security level 3 laboratory facilities at FISABIO-Public Health as follows: After thawing the samples at room temperature, a 200 uL aliquot was taken for RNA extraction, and the remainder was immediately stored at −80°C for the assay in cell lines. RNA extraction was carried out with the fully automated eMAG platform (bioMérieux, France) following the manufacturer's instructions for saliva samples, preceded by lysis with proteinase K (Epicentre) during 20 minutes at 56 °C. Then, the a multiplex RT-qPCR test was performed to detect the SARS-CoV-2 E-gene and the human RNAse-P gene as a sample and extraction control based in the WHO-Charité and U.S. CDC assays [31,33] following the protocol details described by Ferrer et al. [19]. Two replicates per sample of the extracted RNA were performed and virus copies were normalized by mL of saliva.

### SARS-CoV-2 culture from saliva samples in Vero-E6 cells

Vero-E6 cells (ATCC) were cultured in DMEM (Biowest) supplemented with 10% heat-inactivated fetal bovine serum (FBS) (Biowest), 1% penicillin / streptomycin (Biowest) (P/S), 1% non-essential amino acids (Biowest), L-glutamine 200 mM (Biowest) and HEPES 25 mM (Biowest). All cells were incubated at 37°C and 5% CO_2_.

Saliva samples were diluted 1:1 in 1X Dulbecco’s PBS (Gibco) and then centrifuged for 5 minutes at 12,000 g. Three hundred uL of the supernatant was incubated for 1 hour at 37°C with 1.5 × 10^5^ Vero-E6 cells in a 24-well plate in duplicate (Corning). Saliva (including unabsorbed viruses) was then removed and replaced by 500 uL of infection media (DMEM con 2% FBS, 1% penicillin/streptomycin (P/S), 1% non-essential amino acids, 1% L-glutamine, HEPES 25 mM and trypsin TPCK 6ug/mL (Biowest). Cultures were then incubated at 37°C and 5% CO_2_ for 5 days. After 5 days, cytopathic effect (CPE) was assessed by microscopy, recorded as positive or negative [14], and supernatants were collected for RNA extraction following the eMAG platform instructions. A culture was considered positive when the RT-qPCR Ct value in day 5 culture supernatant was < 37 (equivalent to ≥ 2 × 10^3^ SARS-CoV-2 copies/mL). After 5-day culture, CPE was evident in positive controls-cells seeded with 300 uL SARS-CoV-2 stock virus (MAD-6 strain, 1.33 TCID50/mL, CNB-CSIC, Spain)-, absent in negative controls-cells seeded with 300 uL PBS- and variable in the cells seeded with saliva samples, depending on the viral load in saliva. Specifically, a positive CPE was observed in 100% of the controls with an initial number of ≥10^4^ viral genome equivalents, 0% of experiments with an initial number of ≤10^2^ viral genome equivalents and in 80% of experiments with an initial number of viral genome equivalents between 10^2^ and 10^4^.

### Statistical analysis

Considering that each volunteer act as their own control, when comparing the infectious viral load values in saliva at every time with respect to the levels prior to rinsing with the mouthwash, a sample size of five patients per branch was considered sufficient to identify significant differences between groups of more than 20%. Assuming a 10% loss of patients due to abandonment or low viral load and adopting an alpha of 0.05 and a power of 0.8, as five branches were programmed in the trial (CPC, CHX, PVP-I, H_2_O_2_ and the control), 75 patients were the minimum number of individuals to be recruited, who were distributed among the different hospitals.

A Wilcoxon signed-rank test was used to test for mean differences between study time points (basal, 30, 60 minutes) when comparing different treatments (CPC, CHX, PVP-I, H_2_O_2_ and the control) and within each mouthwash. Also, some tests for association between paired samples using Spearman's correlation coefficient were performed to assess relationship between viral load and other clinical continuous variables. All computations and tests were performed using R environment for statistical computing version 3.6.3 and its "stats" package [37].
